# Membrane Lipids’ Metabolism and Transcriptional Regulation in Maize Roots Under Cold Stress

**DOI:** 10.3389/fpls.2021.639132

**Published:** 2021-04-15

**Authors:** Xunchao Zhao, Yulei Wei, Jinjie Zhang, Li Yang, Xinyu Liu, Haiyang Zhang, Wenjing Shao, Lin He, Zuotong Li, Yifei Zhang, Jingyu Xu

**Affiliations:** ^1^Key Lab of Modern Agricultural Cultivation and Crop Germplasm Improvement of Heilongjiang Province, College of Agriculture, Heilongjiang Bayi Agricultural University, Daqing, China; ^2^College of Agriculture, Northeast Agricultural University, Harbin, China

**Keywords:** maize (*Zea mays* L.), lipid metabolism, lipidome, transcriptome, cold stress

## Abstract

Low temperature is one of the major abiotic stresses that restrict the growth and development of maize seedlings. Membrane lipid metabolism and remodeling are key strategies for plants to cope with temperature stresses. In this study, an integrated lipidomic and transcriptomic analysis was performed to explore the metabolic changes of membrane lipids in the roots of maize seedlings under cold stress (5°C). The results revealed that major extraplastidic phospholipids [phosphatidylcholine (PC), phosphatidylethanolamine (PE), phosphatidic acid (PA), and phosphatidylinositol (PI)] were dominant membrane lipids in maize root tissues, accounting for more than 70% of the total lipids. In the transcriptome data of maize roots under cold stress, a total of 189 lipid-related differentially expressed genes (DEGs) were annotated and classified into various lipid metabolism pathways, and most of the DEGs were enriched in the “Eukaryotic phospholipid synthesis” (12%), “Fatty acid elongation” (12%), and “Phospholipid signaling” (13%) pathways. Under low temperature stress, the molar percentage of the most abundant phospholipid PC decreased around 10%. The significantly up-regulated expression of genes encoding phospholipase [phospholipase D (PLD)] and phosphatase PAP/LPP genes implied that PC turnover was triggered by cold stress mainly *via* the PLD pathway. Consequently, as the central product of PC turnover, the level of PA increased drastically (63.2%) compared with the control. The gene-metabolite network and co-expression network were constructed with the prominent lipid-related DEGs to illustrate the modular regulation of metabolic changes of membrane lipids. This study will help to explicate membrane lipid remodeling and the molecular regulation mechanism in field crops encountering low temperature stress.

## Introduction

Maize (*Zea mays* L.), originated from subtropical zones, is a typical thermophilic crop that requires a relatively high growth temperature, especially in the seedling and vegetative growth stage (Rodríguez et al., 2014). In northeastern China (around 44° north latitude), low temperatures in early spring and sudden temperature drops in late spring seriously affects the growth and development of maize seedlings.

Understanding the response of plants to low temperature stress at the molecular level is of great importance in developing cold-resistance crops. The plant membrane is the first barrier to cope with external environmental stimuli, which could be attributed to its typical fluidity and certain protective properties (Chinnusamy et al., 2007). When plants are exposed to low temperatures, the fluidity of the plant cell membrane will be improved, which will increase the tolerance of the plant to low temperatures (Chinnusamy et al., 2007; Gao et al., 2015). The alteration of membrane glycerolipids composition and saturation has been considered as a major strategy for plants to respond to temperature stress (Zheng et al., 2011; Barrero-Sicilia et al., 2017). Low temperature stress increased the unsaturation of fatty acids, which increased the fluidity of the plant membrane, reduced the tendency of non-bilayer phase formation, and enhanced the integrity and function of the plant membrane (Uemura and Steponkus, 1997; Hou et al., 2016).

Glycerolipids are essential components of the plant membrane that include extraplastidic phospholipids such as phosphatidylcholine (PC), phosphatidylethanolamine (PE), phosphatidic acid (PA), phosphatidylinositol (PI), and phosphatidylserine (PS), and plastidic lipids such as monogalactosyldiacylglycerol (MGDG), digalactosylmonoacylglycerol (DGMG), phosphatidylglycerol (PG), and sulfoquinovosyldiacylglycerol (SQDG). Among the extraplastidic phospholipids, PC is the most abundant lipid in eukaryotic cells, which accounts for 25–60% of non-plastidic membrane lipids in plants (Ohlrogge and Browse, 1995; Li-Beisson et al., 2010). In *Arabidopsis*, PC was initially established by transferring P-choline from CDP choline to *sn-1*,*2*-diacylglycerol (DAG) (Tasseva et al., 2004). PA is the simplest glycerophospholipids in structure, yet it is a very important phospholipid class. Although the proportion of PA is not large, it is an important signaling lipid, and is also known as the key precursor for the synthesis of major glycerophospholipids and glyceroglycolipids (Dubots et al., 2012). Glycerolipids contain various molecular species, in which the length of acyl chains and the number of acyl double bonds at *sn-1* and *sn-2* position are different, and lipid remodeling occurs at different developmental stages or under non-optimal growth conditions (Testerink and Munnik, 2011).

An increasing number of studies have shown that membrane lipids’ metabolism plays important roles in the temperature stress response of plants (Li et al., 2016; Narayanan et al., 2016; Gu et al., 2017). Lipids’ remodeling and the enzymes involved in related processes are particularly critical for plants to adapt to cold environments (Li et al., 2016; Gu et al., 2017). The phospholipase PLD (phospholipase D) was activated under low temperature, which led to an increased accumulation of lipid signaling molecules PA (Ruelland et al., 2002). The content of PA can increase rapidly and maintain a high level only for a few minutes after low temperature exposure (Peppino Margutti et al., 2017). The resistance of *Arabidopsis* PLD mutant to cold was decreased, which might be attributed to the reduced PA content (Li et al., 2004). In *Brassica napus*, the unsaturation of PC and MGDG increased gradually under a low temperature (Tasseva et al., 2004). In *Arabidopsis*, the percentage of PA (34:6) was elevated under a low temperature (Zheng et al., 2016).

The expression of lipid-metabolism-related genes has been suggested to be associated with low temperature response in plants. In rice, over-expression of *AtGPAT* enhanced the unsaturated fatty acid content of PG and increased cold tolerance (Li et al., 2018). In *Arabidopsis*, the *Atdgat1* mutants were found to be more sensitive to chilling and freezing stresses compared with wild-type plants (Tan et al., 2018). The expression of *AtDGK2* is induced in various tissues under low temperature stress, which plays an important role in the cold signaling process (Gómez-Merino et al., 2004). The knock-out of *OsFAD8* further reduced membrane fluidity in rice under cold stress (Tovuu et al., 2016).

In a previous report, the lipid metabolism in leaves of maize seedlings under low temperature stress was elaborated by our group, which showed that maize was an 18:3 plant and cold stress exerted significant impacts on membrane lipids’ metabolism (Gu et al., 2017). Since the knowledge of lipids’ metabolism in root tissues is limited, the lipidomic and transcriptomic analysis of maize roots under low temperature stress was conducted in this study. This study will provide an overall understanding of the lipid metabolism in maize seedlings in adaptation to low temperature stress.

## Materials and Methods

### Plant Growth, Treatments, and Sampling

The inbred line He344, a major maize variety planted in Northeast China, was used as experimental material. Maize seeds of the same size were selected and disinfected with 10% NaClO for 30 min. After repeated washing with distilled water, the seeds were put into a 25°C incubator for dark germination. After germination, maize seedlings were cultured with 1/2 Hoagland nutrient solution (pH = 5.5) in a growth chamber. Hoagland nutrient solution was used for precise temperature control and sampling of root tissues. The temperature of the chamber was set at 22°C, with 16/8 h (light/dark) photoperiodic cycle. About half of the 2-week-old maize seedlings were moved into a 5°C chamber, and the rest of the seedlings were kept in the 22°C growth chamber as control. Maize root samples were collected 3 days after cold treatment, and each sample had at least three replicates. The collected samples were wrapped in silver paper quickly, rapidly put into liquid nitrogen for freezing, and then stored at −80°C.

### RNA-seq Analysis and qRT-PCR Validation

Total RNA was extracted from root tissues of 2-week-old maize seedlings after 3 days under 5°C treatment (samples from 22°C growth chamber were used as control) using TRIzol reagent (Invitrogen). The purity and concentration of RNA samples were examined, and then the library was constructed. After the database was qualified, it was sequenced by Illumina platform. The RNA-seq data were mapped to the maize reference genome B73 RefGen_v3.

Fragments Per Kilobase of exon model per Million mapped reads (FPKM) of each gene were measured from the length of the gene and reads count mapped to the genes. The counting of the read numbers mapped to each gene was performed by HTSeq v0.6.1. The screening of DEGs (differentially expressed genes) was performed using the Bioconductor package “edgeR” in R among the treatment samples. DEG parameters were set at false discovery rate (FDR) < 0.01 and | Log2 fold-change| ≥ 1. The KOBAS (v2.0.12) software was used to test the statistical enrichment of the DEGs in the Kyoto Encyclopedia of Genes and Genomes (KEGG) pathways, and a modified *P*-value (*q*-value) ≤ 0.05 was the criteria for significantly enriched KEGG pathways.

In order to further validate the reliability of the RNA-seq results, qRT-PCR analysis was implemented. The cDNA was compounded using the ReverTra Ace qPCR RT Master Mix (TOYOBO, Osaka, Japan). Real-time quantitative RT-PCR was accomplished in 96-well plates with a SYBR Select Master Mix RT-PCR system. *ZmACTIN* and *ZmGAPDH* were used as internal control, and the qRT-PCR primers are listed in Supplementary Table 1. The results of qRT-PCR were reckoned by 2^–Δ^ ^Δ^ ^ct^ method, and the data from the maize samples grown in 22°C were used as the calibrators (Czechowski et al., 2005; Zhang et al., 2014).

#### Membrane Lipid Extraction and Analysis

The method was modified according to a previous report (Narayanan et al., 2016). A total of 3 ml isopropanol (0.01%BHT) was added to a 50 ml glass tube and the glass tube was placed in the nitrogen blowing instrument and preheated to 75°C. About 200 mg maize root samples were rapidly added to the preheated glass tube and kept at 75°C for 15 min. Distilled water (0.6 ml) and chloroform (1.5 ml) were added to the tube, vortexed, and shaken for 1 h in a shaking table, and then the extract was transferred to a new glass tube. Next, 4 ml chloroform: methanol (2:1) mixture was added to the glass tube, vortexed, and shaken for 30 min on a shaking table. The extraction procedure was repeated 3–4 times, and then the mixed liquids were compounded and washed with KCl (1 ml). The upper liquid was discarded, and the remaining liquid was blown to full evaporation with a nitrogen blowing instrument, and then stored at −20°C. Lipids were analyzed by Electrospray Ionization-Mass Spectrometry (ESI-MS/MS), which was accomplished at Kansas Lipidomics Research Center (KLRC, United States).

The precursor (Prec) and neutral loss (NL) scans were applied to obtain polar lipid profiles. The samples were introduced into the electrospray ionization source for further generation of lipid molecular ions, including PC, lysoPC, PE, and lysoPE positive [M + H]^+^ ions, MGDG, DGDG, PG, PI, PA, and PS positive (M+NH4)^+^ ions, and lysoPG negative (M−H)]^–^ ions. A series of peak values of lipid content were detected by electrospray ionization. The peaks on the spectra were quantified in comparison to a group of internal standards. The data for each lipid molecular species were normalized and displayed as mol% of the total lipids analyzed.

### Co-expression Analysis of Lipid Related DEGs

Pearson correlation coefficient was calculated according to the expression data of DEGs (differentially expressed genes, | Log2FC| ≥ 1). After removing the self-pairing and duplication, the relevant cut-off value of 0.9 was applied, and the co-expression network was constructed with the reserved gene pairs. The constructing and visualizing of the co-expression network was carried out with the Cytoscape software.

### Statistical Analysis

All statistical analyses were conducted with SPSS statistics 19.0 (SPSS Inc.), and significance levels of the data were calculated by Student’s *t*-test method. ^∗^*P* < 0.05 and ^∗∗^*P* < 0.01 represent different significance levels.

## Results

### Changes of Membrane Lipids in Maize Roots Under Cold Stress

To explore the changes of membrane lipid species in the roots of maize seedlings under low temperature conditions, polar lipids extracted from maize roots samples under 5°C (and 22°C control) were analyzed by lipidomic approach. A total of 12 different types of lipids were detected, including six phospholipid classes (PC, PE, PA, PI, PS, and PG), three classes of *lyso*-phospholipids (LPC, LPE, and LPG), two galactolipids (MDGD and DGDG), and one sulfolipid (SQDG).

**FIGURE 1:**
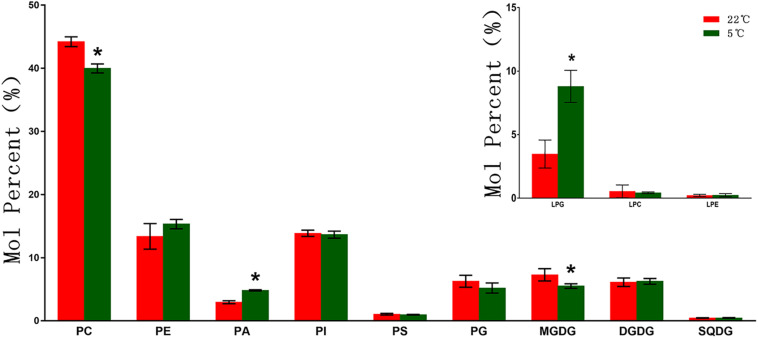
Changes of glycerolipids species in maize roots under cold stress (5°C). PC, phosphatidylcholine; PE, phosphatidylethanolamine; PA, phosphatidic acid; PI, phosphatidylinositol; PS, phosphatidylserine; PG, phosphatidylglycerol; MGDG, monogalactosyldiacylglycerol; DGDG, digalactosyldiacylglycerol; SQDG, sulfoquinovosyldiacylglycerol; LPC, *Lyso*-PC; LPA, *Lyso*-PA; LPG, *Lyso*-PG. Values (mol%) are means 5 ± standard deviation (SD) (*n* = 5). “*” indicated that the value was significantly different from the control (*P* < 0.05).

As shown in Figure 1, phospholipids are the main membrane lipids in maize root tissues, accounting for more than 70% of the total lipids. Among all phospholipids, PC is the most abundant lipid, accounting for around 40% of the total lipids, while the remaining phospholipids account for about 30%. Under low temperature (5°C) stress, the molar percentage of PC decreased around 10% (9.5%) compared with the control (22°C). A significantly enhanced accumulation of PA was observed under cold treatment, with a 63.3% increase compared with the control. The molar percentage of PE was found increased by 14.5%, whereas the proportion of PI and PS were not altered significantly. The level of the exclusive plastidic phospholipid PG was also increased, which was 17.1% higher than the control. The galactolipids MGDG and DGDG each accounted for about 6% of the total polar lipids, while the sulfolipid SQDG content was extremely low. Under low temperature stress, the content of MGDG decreased significantly, while DGDG and SQDG increased slightly. Under low temperature stress, the level of *lyso*-phospholipids altered to varying degrees, and the content of LPG increased more than 1.5 times in comparison with the control.

### Alterations in Molecular Species of Membrane Lipids in Maize Roots Under Cold Stress

**FIGURE 2:**
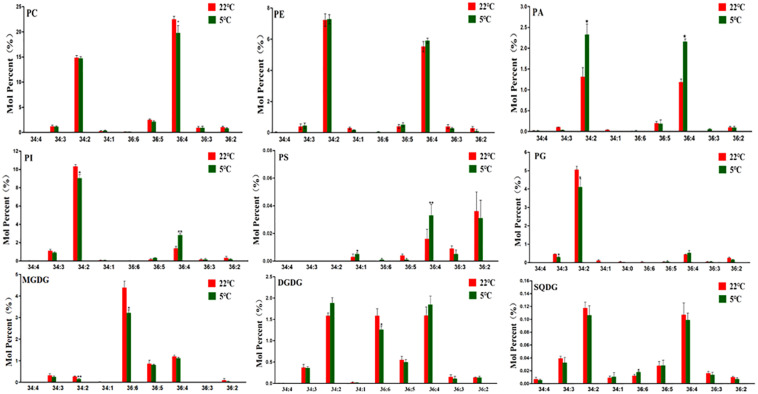
Changes in molecular species of major membrane lipids in maize roots under cold stress (5°C). PC, phosphatidylcholine; PE, phosphatidylethanolamine; PA, phosphatidic acid; PI, phosphatidylinositol; PS, phosphatidylserine; PG, phosphatidylglycerol; MGDG, monogalactosyldiacylglycerol; DGDG, digalactosyldiacylglycerol; SQDG, sulfoquinovosyldiacylglycerol. Values (mol%) are means 5 ± standard deviation (SD) (*n* = 5). “*” indicated that the value was significantly different from the control (*P* < 0.05). “**” Indicated that the value was extremely different from the control.

The molecular species of lipids samples from maize roots were analyzed by ESI-MS/MS and were presented as the numbers of carbon atoms and double bonds (total number of carbon atoms: total number of double bonds) of two fatty acid chains on each lipid class. The molecular species in phospholipids are dominated by C34 and C36 species, of which C34:2 and C36:4 account for a relatively high proportion in phospholipids (Figure 2). Compared with the control, C34:2 had no significant changes, while the content of C36:4 significantly decreased 11.9% in PC. In the plastidic phospholipid PG, the molar percentage of C34:2 decreased by 18.7%, while that of C36:4 increased 19.9% compared with the control. In PA, the molar percentage of C34:2 and C36:4 both increased significantly. The levels of C34:2 and C36:4 altered with an opposite trend in PI under cold treatment. In PS, the molar percentages of C34:1, C36:4, and C36:6 were all raised under cold stress, while the molar percentage of C36:2 decreased compared with the control (Figure 2).

As shown in the lower panel of Figure 2, C36 molecular species are dominant in galactolipids MGDG, which is consistent with the previous finding that maize is an 18:3 plant (Gu et al., 2017). Under low temperature stress, the molar percentage of C36:6 MGDG was significantly lowered than that of the control. In DGDG, the percentage of C36:6 and C36:5 molecules decreased under cold stress.

### Transcriptomic Analysis of Maize Roots Under Cold Stress

To investigate the transcriptional regulation of the membrane lipids’ metabolism in maize roots under cold stress, the transcriptomic analysis was conducted. A total of 55.14 GB clean data were produced by six runs; the clean data of each run reached 8.14 GB at least. The percentage of Q30 base was 94.52%, and it was mapped to the maize reference genome (B73 RefGen_v3). The principal components analysis (PCA) showed that the three replicates of each sample were clustered together, which indicates that the three repeats of the same sample are consistent (Supplementary Figure 2).

**FIGURE 3:**
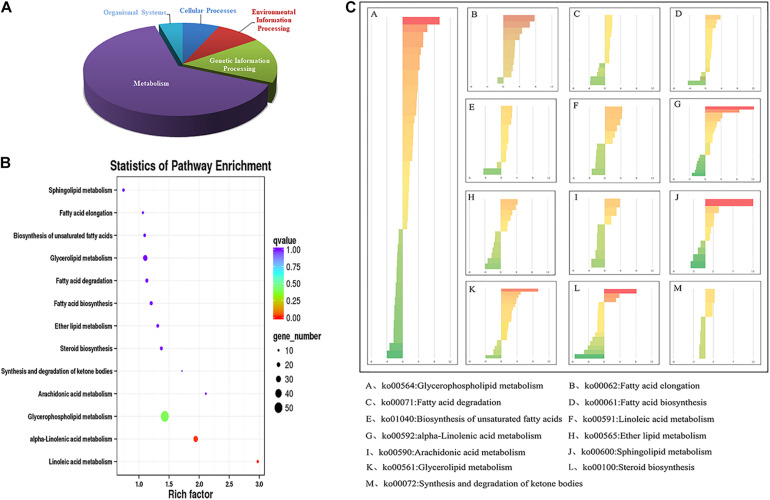
Lipid-related DEGs annotated in KEGG pathways under cold stress (5°C). DEGs, differentially expressed genes; KEGG, Kyoto Encyclopedia of Genes and Genomes. **(A)** The distribution of metabolism-related DEGs identified in the maize roots transcriptome; **(B)** the statistics of KEGG pathway enrichment of DEGs related to lipid metabolism; **(C)** the DEGs involved in different lipid metabolic pathways. The number of the genes in each category were displayed on the *x*-axis.

As shown in Figure 3, in the comparison group (5 vs 22°C), a total of 2769 DEGs were annotated in KEGG pathway, and were classified into five categories. The results showed that most of the DEGs were enriched in the metabolic processes (Figure 3A), which implied the metabolic processes were greatly affected under cold stress.

In the metabolic pathway, a total of 208 DEGs were annotated in lipid metabolic pathways, and most of them were involved in fatty acid metabolism, suggesting that those pathways were significantly influenced under cold stress (Figure 3B). The lipid-related DEGs were categorized into 13 pathways as shown in Figure 3C, and the most enriched pathway was the “Glycerophospholipid metabolism” pathway. There were 45 DEGs in the “Glycerophospholipid metabolism pathway,” including 28 up-regulated and 17 down-regulated DEGs, respectively. In the “Fatty acid elongation” and “Biosynthesis of unsaturated fatty acids” pathway, most genes were up-regulated (Figure 3C).

### Differential Responses of Lipids Related DEGs in Maize Roots Under Cold Stress

Maize lipid-related genes were further screened from maize root transcriptome data according to the previously published *Arabidopsis* lipids-related gene databases (Beisson et al., 2003; Troncoso-Ponce et al., 2013). A total of 189 lipid-related DEGs were annotated and recruited, most of which were involved in the “Eukaryotic phospholipid synthesis” (12%), “Fatty acid elongation” (12%), and “Phospholipid signaling” (13%) pathways (Supplementary Figure 2).

**FIGURE 4:**
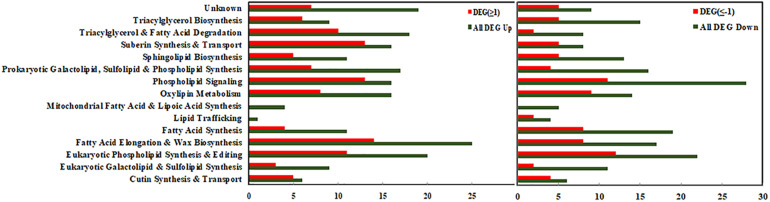
Functional categorization of lipid-related genes from maize roots transcriptome under cold stress (5°C). **Left** columns represent up-regulated genes, and **right** columns represent down-regulated genes. In each category, the green colored column represents the total differentially expressed genes (DEGs), and the red colored column represents the significantly differentially expressed genes (DEGs with Log2FC ≥1 or ≤–1). The number of the genes in each category were displayed on the *x*-axis (the ones on the upper *x*-axis indicate the DEGs with Log2FC ≥1 or ≤–1).

In order to demonstrate the differential regulation of lipid-related DEGs in each metabolic pathway, the bar chart representing the up/down regulated DEGs is shown in Figure 4. The left panel represents up-regulated genes, and the right panel represents down-regulated genes. In each category, the green columns represent the total DEGs, and the red columns represent the significant DEGs (Log2FC ≥ 1 or ≤−1). As shown in Figure 4, most of the DEGs involved in “Phospholipid signaling,” “Fatty acid elongation,” “Eukaryotic phospholipid signaling,” and “Triacylglycerol & Fatty acid degradation” pathways were significantly up-regulated, and the number of the significantly up-regulated DEGs (Log2FC ≥ 1) was among 10–14. A number of DEGs involved in “Eukaryotic galactolipid & Sulfolipids synthesis,” “Phospholipid signaling,” and “Oxylipin metabolism” were found significantly down-regulated (Log2FC ≤ −1), which demonstrated differential regulation of major lipids’ metabolism pathways under cold stress.

### Analysis of Significant DEGs Involved in Lipid Metabolism Under Cold Stress

**FIGURE 5:**
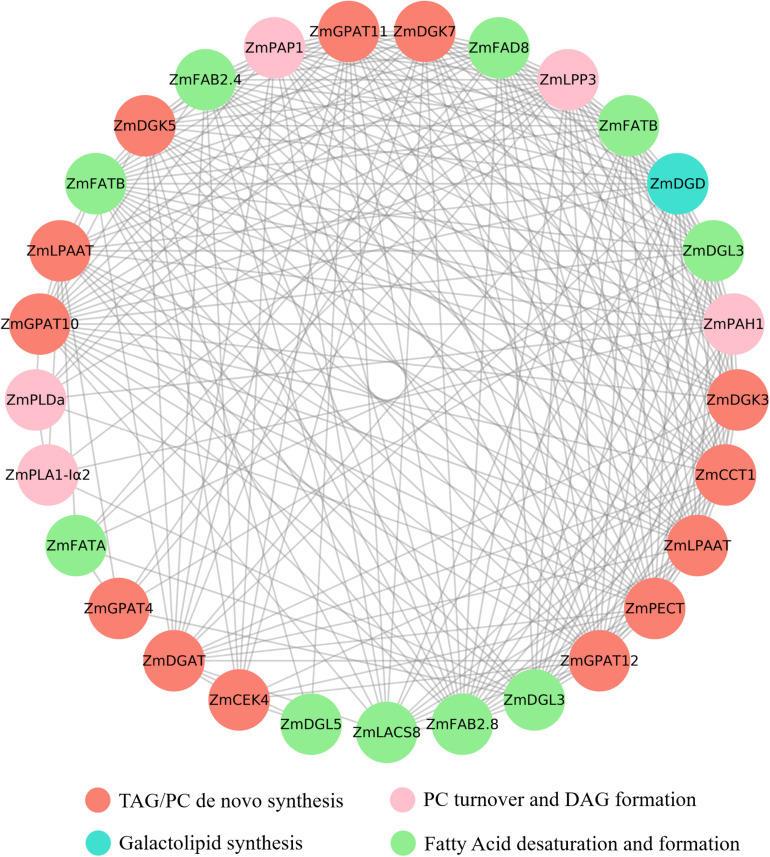
Co-expression of lipid related genes. Different colors represent different genes. The co-expression network was constructed with the reserved gene pairs. The constructing and visualizing of the co-expression network was carried out with the Cytoscape software.

Among the lipid related DEGs, some significantly up-regulated DEGs were involved in the major lipid metabolism pathway. As shown in Figure 5, in the endoplasmic reticulum (ER) TAG (triacylglycerol) *de novo* synthesis pathway (the Kennedy pathway), genes encoding glycerol-3-phosphate acyltransferase (GPAT) and lysophosphatidic acyltransferase (LPAAT) were significantly up-regulated under low temperature, including four GPATs (the most up-regulated isoform GRMZM2G070304, Log2FC = 9.28), two LPAAT (GRMZM2G079109, Log2FC = 3.43), and one DGAT1 (GRMZM2G130749, Log2FC = 2.09). In the PC and PE *de novo* biosynthesis pathway, the related genes were also significantly up-regulated, including CCT (choline phosphate cytidylyltransferase, Log2FC = 2.69) and CEK (choline kinase, Log2FC = 1.11). One DEG encoding PAH (PA phosphatase) was significantly up-regulated (GRMZM2G099481, Log2FC = 4.27).

**FIGURE 6:**
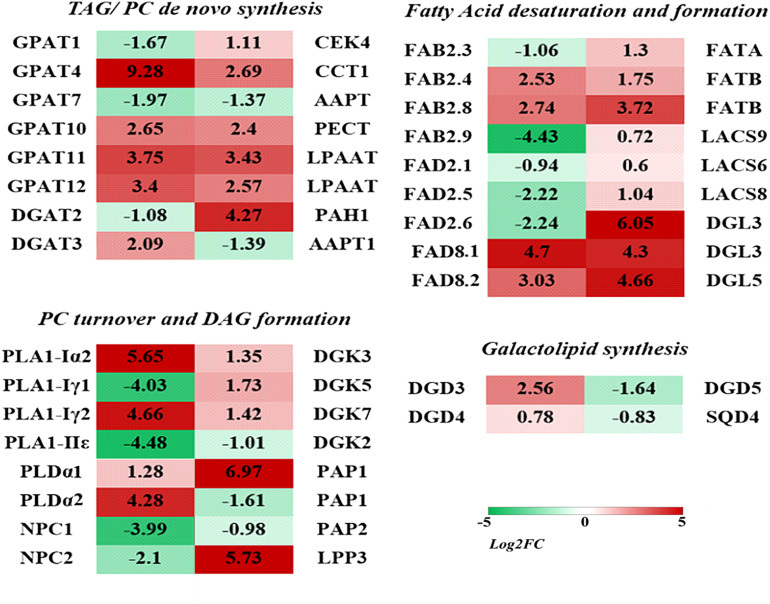
Differentially expressed genes (DEGs) involved in major lipid metabolism pathways in maize roots under cold stress (5°C). The heatmaps were constructed to illustrate the differential expression profiles of significant lipid related DEGs. The number in each color block represents the Log2(fold-change) of the corresponding genes, and the negative number represents down-regulated DEGs. The color scale was provided. Red color indicates higher expression level, and green color indicates lower expression level.

Phosphatidylcholine and PE could be hydrolyzed by PLD and non-specific phospholipase C (NPC) to generate PA and DAG. In the “PC turnover and DAG formation” process, two PLD (GRMZM2G054559 and GRMZM2G179792) were found significantly up-regulated (Log2FC ¿ 4) under low temperature stress, while two NPC were found significantly down-regulated (Figure 6 and Supplementary Table 2). Another major pathway for PC degradation is mediated by phospholipase A (PLA) to form phospholipase C (PLC). Two PLA1 genes were drastically up-regulated (Log2FC is 4.66 and 5.65), which suggested enhanced degradation of PC under cold stress. Monogalactosyldiacylglycerol synthase (MGD) and digalactosyldiacylglycerol synthase (DGD) are key enzymes responsible for the biosynthesis of plastidic galactolipids MGDG and DGDG. Under low temperature stress, two DGDs were up-regulated, and the most significant one had a Log2FC of 2.56.

The synthesis of fatty acids is accomplished in plastids. A number of genes involved in the *de novo* synthesis and desaturation of fatty acids were up-regulated under cold stress, including FAB2, FAT, and LACS. A set of fatty acid desaturase (FADs) were also involved in the fatty acid desaturation in both phospholipids and galactolipids metabolism pathways. Under low temperature stress, two genes encoding FAD8 were significantly up-regulated, and the Log2FC was 4.66 and 5.65, respectively. A group of lipases that catalyze the hydrolysis of phospholipids and galactolipids to release free fatty acids were also up-regulated, including three diacylglycerol lipase (DGLs) (Log2FC is 4.3 and 6.05).

To verify the gene expression in transcriptome data, ten lipid-related genes were selected for quantitative RT-PCR analysis. The results showed that under low temperature stress, the expression of genes such as ZmLPP, FATB, and ZmFAD8 increased significantly, which is consistent with the trends in transcriptome data (Supplementary Figure 3).

### The Co-expression Analysis of Lipid-Related Genes

**FIGURE 7:**
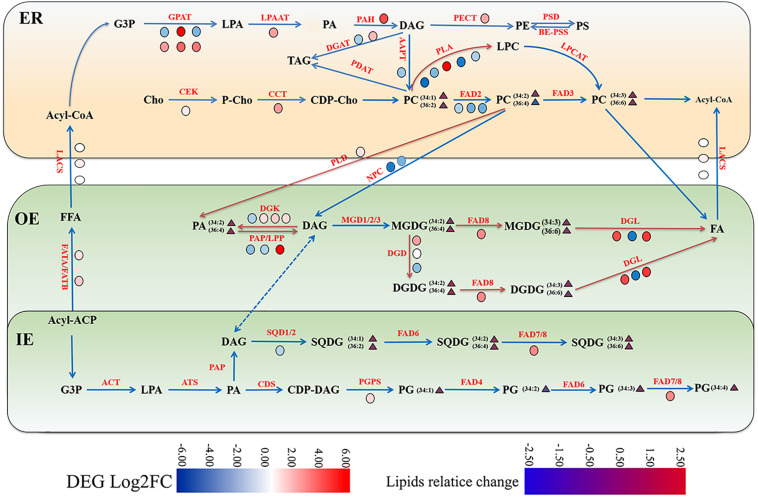
Gene-metabolite network illustrating membrane lipid metabolism in maize under cold stress. The glycerolipids synthesis pathways were depicted and the involved genes and lipid metabolites were symbolized. The relative change of lipid molecular species and the relative expression levels of selected genes were marked as heatmap icons. The color scale is provided. Red color indicates higher expression level, and green color indicates lower expression level. The red arrows represent activated steps by cold. ER, endoplasmic reticulum; OE, outer envelope. IE, inner envelope.

Pearson correlation coefficient was calculated according to the expression data of DEGs, and the constructing and visualizing of the co-expression network was carried out with the Cytoscape software. A total of 62 lipid-related genes involved in different lipids’ metabolic processes were screened in the transcriptome data, and the co-expression analysis was performed. The results indicated that the expression of 19 genes was closely correlated (Figure 7). Most of the genes were co-expressed with multiple genes. The expression of *ZmGPAT10* (GRMZM2G020320) was found to be correlated with 22 genes, including *ZmPAP1* (GRMZM2G024144, *r* = 0.96), *ZmFAD8* (GRMZM2G074401, *r* = 0.99), *ZmDGD* (GRMZM2G092588, *r* = 0.99), and *ZmPETC* (GRMZM2G155357, *r* = 0.99).

The transcriptome data was screened using the online transcription factor database^1^. A total of 42 transcription factor families were identified, comprising of 553 transcription factors, among which the most abundant transcription factor families were SLD, WRKY, and NAC (Supplementary Figure 4).

A co-expression analysis among transcription factors and lipid-related genes was conducted and revealed that there were 201 TFs related to the TAG/PC *de novo* synthesis pathway. Among them, important regulatory genes related to lipid biosynthesis and seed development were discovered, including AP2/ERF(25), bHLH(14), B3(5), and MYB(12). At the same time, there are 110 TFs related to PC turnover and DAG formation, and 174 TFs related to fatty acid desaturation and formation, which all contain different numbers of TFs, such as AP2/ERF, bHLH, B3, and MYB. This result indicated that these genes might play important roles in lipid metabolism in coping with cold stress (Supplementary Figure 5).

### The Gene-Metabolite Network of Lipids Metabolism in Maize Roots Under Cold Stress

Based on the combined analysis of the transcriptomic and lipidomic data, a scenario diagram was constructed to illustrate the gene-metabolite network. As shown in Figure 7, the metabolic pathways of glycerolipids were described, and the differential gene expression profiles and lipid changes were marked with colored heatmap icons.

Phosphatidylcholine is the most abundant phospholipids in maize root tissues, which is initially synthesized by transferring P-choline from CDP-choline to DAG in ER. The *de novo* production of DAG is through the Kennedy Pathway catalyzed by GPAT, LPAT, and PAH, which were all up-regulated at the transcriptional level under low temperature stress as observed in the cold transcriptome. The ER generated PC is also an essential precursor to generate PA and DAG. Both PLD and NPC were involved in hydrolyzing PC (and/or PE) to produce PA and DAG. Under low temperature treatment, two PLD genes were found significantly up-regulated, whereas two NPCs were found down-regulated (Figure 6 and Supplementary Table 2), which might suggest that the PLD pathway was responsible for PC turnover in maize roots under cold stress. Furthermore, two genes encoding PLA1, which mediates PC degradation to form PLC, were drastically up-regulated. These findings suggested enhanced PC turnover under cold stress, which might explain the decreased PC content as revealed by lipidomic analysis. The enhanced accumulation of PA could be attributed to the activated PC hydrolyzation *via* PLD pathway.

The membrane lipids in plastid/chloroplasts have a distinct composition, which are dominated by galactolipids MGDG and DGDG. Under low temperature conditions, one DGD1 and FAD8 (Figure 7), which were involved in DGDG synthesis and desaturation, respectively, were obviously up-regulated. However, the molar percentage and unsaturation of DGDG did not change a lot in metabolic aspects. The plastidic lipids MGDG decreased significantly under cold stress, which might be due to the enhanced conversion to DGDG by the action of DGD, and both DGDG and MGDG could undergo degradation through the cold triggered DGLs.

## Discussion

**FIGURE 8:**
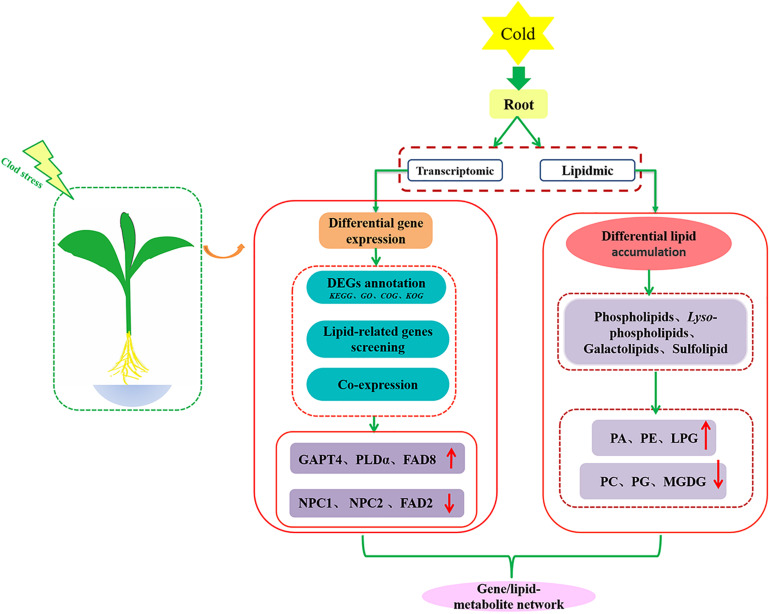
Schematic diagram of transcriptomic and lipidomic analysis strategies.

Membrane lipid metabolism and remodeling are key strategies for plants to cope with temperature stresses (Moellering et al., 2010; Li et al., 2016). In a previous study, we provided evidence that maize is an 18:3 plant with dominating C36:6 (two 18: 3 acyl chains) molecular species in their galactolipids DGDG and MDGD (Gu et al., 2017). In this study, a combined lipidomic and transcriptomic strategy was used to explore the lipidomic changes and the transcriptional regulation in root tissues of maize seedling under cold stress. As shown in Figure 8, the changes of glycerolipids profiles, the differential expression of lipid-related genes, and co-expression network of transcription factors and lipid genes were interactively investigated.

A number of lipidomic studies have shown that membrane glycolipid profiles are largely influenced by cold stress, however, most of the research was conducted in the above-ground tissues of the plants (Degenkolbe et al., 2012; Zheng et al., 2016). The extraplastidic phospholipids classes (PC, PE, PA, and PI) are the most abundant lipid species. Under low temperatures, a significant decrease of PC was observed. In the parallel transcriptomic analysis, the eukaryotic PC *de novo* biosynthesis pathway and PC degradation and signaling pathway were activated by cold stress as manifested by the up-regulation of genes involved in those pathways. Nevertheless, the dropped PC level suggested that PC degradation mediated by PLD and PLA was dominant under low temperature stress. PC is known as the major “bilayer lipid,” which is crucial in maintaining the membrane integrity under stress conditions (Li et al., 2015; Lin et al., 2016; Zheng et al., 2016). The reduction of PC under cold stress might result in a certain degree of membrane damage in plant cells.

Phosphatidic acid is the major central lipids’ intermediate and signaling molecule. In addition to the PA generated through the phosphorylation of DAG by PAH in the ER, a large proportion of PA could be produced by hydrolysis of phospholipids, including PC and PE, through the PLD and PLC/DGK pathways (Laxalt and Munnik, 2002; Ruelland et al., 2002; Arisz et al., 2009; Benning, 2009). PA plays important roles in a variety of cellular processes involving plant growth, reproduction, and signal transduction under abiotic stresses (Ruelland et al., 2015; Meringer et al., 2016; Tan et al., 2018). Previous research had shown that the increase of PA content was beneficial in reducing the damage of reactive oxygen species to *Arabidopsis* under cold stress (Moellering et al., 2010; Li et al., 2016). In this study, we found that the accumulation of PA significantly improved in maize roots under cold stress, which suggested that the increase of PA content may help to alleviate the damage of maize seedlings under cold stress. As illustrated in the gene-metabolite network (Figure 7), the up-regulated PLD genes and down-regulated NPCs suggested that the PLD pathway was responsible for PC turnover in maize root under cold stress. In a previous study, we found that PA produced by the action of PLD was significantly enhanced and contributed to the plastidic lipids’ synthesis in maize leaves under cold stress (Gu et al., 2017). These findings implied that generation of PA *via* the PLD pathway was triggered by cold in both above-ground and under-ground tissues of maize seedlings.

In plants, the unsaturation PG was considered to be closely related to low temperature response (Mikami and Murata, 2003). In tobacco, the increase of saturated fatty acid level of PG makes plants sensitive to cold stress (Mikami and Murata, 2003). Fatty acid desaturases FAD8 and catalyzes the desaturation of FAs that are esterified to PG and result in high linolenate (18:3) lipid species (Zhao et al., 2019). The knocked-out *FAD8* led to reduced membrane fluidity in rice under low temperature stress (Tovuu et al., 2016). In this study, two genes encoding FAD8 annotated in the transcriptomic data (*ZmFAD8.1* and *ZmFAD8.2*) were significantly up-regulated under low temperature stress. Moreover, in the parallel lipidomic analysis, an obvious increase in 36:4 species was observed. This result indicates that the increased unsaturation of PG may be beneficial to the adaptation of cold stress for the root tissue of maize seedlings.

Membrane lipid remodeling occurs when plants encounter cold stress (Chen and Thelen, 2016). Previous studies have shown that, as the growing temperature deceased, the content of DGDG increased and the ratio of MGDG/DGDG decreased, which could help to enhance the cell membrane stability under stress (Campos et al., 2003; Moellering et al., 2010). In this study, a significant decrease of MGDG and a slight increase of DGDG was observed, resulting in a large reduction in the MGDG/DGDG ratio in maize seedlings under cold stress. MGD catalyzes the galactose transfer from UDP-galactose to DAG framework to form MGDG, and then the second galactose is diverted from UDP-galactose to MGDG by DGD for the final formation of DGDG (Wang et al., 2020). In our transcriptomic data, the expression of two maize *DGDs* were up-regulated, and the most apparent one had a Log2FC of 2.56.

A number of reports have indicated that the over-expression of DGAT and DGK could regulate the dynamic balance of DAG, PA, and TAG in *Arabidopsis* under cold stress (Moellering et al., 2010; Li et al., 2016). Over-expression of AtDGAT1 enhances cold tolerance of *Arabidopsis* (Arisz et al., 2018). AtDGAT1, AtDGK2, AtDGK3, and AtDGK5 were shown to be responsible to cold stress tolerance in *Arabidopsis* (Tan et al., 2018). In this study, the up-regulation of a member of DGAT and DGK isoforms suggested DAG-TAG and DAG-PA pathways were activated under cold stress, which may contribute to cold adaptation of maize seedlings. The accumulation of TAG is influenced by a series of transcription factors, including WRI1, MYB, and bZIP (Song et al., 2013; Liu et al., 2014; Chen et al., 2020). Researchers have shown that the over-expression of *AtWRI1* gene in maize and rapeseed enhanced TAG production (Cernac and Benning, 2004; Shen et al., 2010). In this study, two *ZmWRI1* genes (GRMZM2G124524, Log2FC = 1.88 and GRMZM2G174834, Log2FC = 2.62) were up-regulated under cold stress. The activation of TAG biosynthesis in maize roots under low temperature stress might be attributed to increased fatty acids derived from hydrolytic enzymes, and these excess FAs may be temporarily stored in TAG.

## Conclusion

In summary, we observed active changes of membrane lipids in maize roots under cold stress, including decreased PC and increased PA contents, and the enhanced transcription of a set of lipid-related genes. The results revealed the activation and interaction of phospholipid and galactolipid synthesis pathways in response to cold, and the modular regulation of metabolite accumulation and gene expression in the respective processes. It should be noted that this model is based on combined analysis of transcriptomic and lipidomic changes, and the possibility of posttranscriptional regulation on proteins/enzymes could not be excluded. Since information on the regulation of lipid metabolism in 18:3 plants is still lacking, there are still a great deal of unanswered questions that need further investigation.

## Data Availability Statement

The original contributions presented in the study are publicly available. This data can be found here: NCBI repository, accession numbers SRR13312968, SRR13312969, SRR13312971, and SRR13312972.

## Author Contributions

XZ and YW performed the experiments and prepared the manuscript. JZ, LY, XL, and WS prepared the samples. HZ, LH, and ZL analyzed the data. JX and YZ conceived the experiments and revised the manuscript. All authors contributed to the article and approved the submitted version.

## Conflict of Interest

The authors declare that the research was conducted in the absence of any commercial or financial relationships that could be construed as a potential conflict of interest.

## Footnotes

1 http://planttfdb.cbi.pku.edu.cn/

## Supplementary Material

Supplementary Figure 1 Quality inspection of maize seedling root transcriptome data under low temperature stress.

Supplementary Figure 2 Functional annotation of genes related to lipid metabolism in maize roots under low temperature stress.

Supplementary Figure 3 Verification of differentially expressed genes in maize seedling roots under low temperature stress by qRT-PCR.

Supplementary Figure 4 Enrichment of transcription factors of genes related to lipid metabolism in maize roots under low temperature stress.

Supplementary Figure 5 Co-expression analysis of transcription factors and lipid-related genes.

Supplementary Table 1 Related genes qRT-PCR verification primers.

Supplementary Table 2 Expression of lipid-related genes under cold stress.

## Abbreviations

PC: phosphatidylcholine
PE: phosphatidylethanolamine
MGDG: monogalactosyldiacylglycerol
DGDG: digalactosyldiacylglycerol
SQDG: sulfoquinovosyldiacylglycerol
PI: phosphatidylinositol
PE: phosphatidylethanolamine
PS: phosphatidylserine
PA: phosphatidic acid
DAG: diacylglycerol
PLD: phospholipase D
PLC: phospholipase C.
