# Supplementary figures and images for: Membrane Lipids’ Metabolism and Transcriptional Regulation in Maize Roots Under Cold Stress
Source: Front Plant Sci. 2021 Apr 15;12:639132. doi: 10.3389/fpls.2021.639132 (PMC8083060; doi:10.3389/fpls.2021.639132)

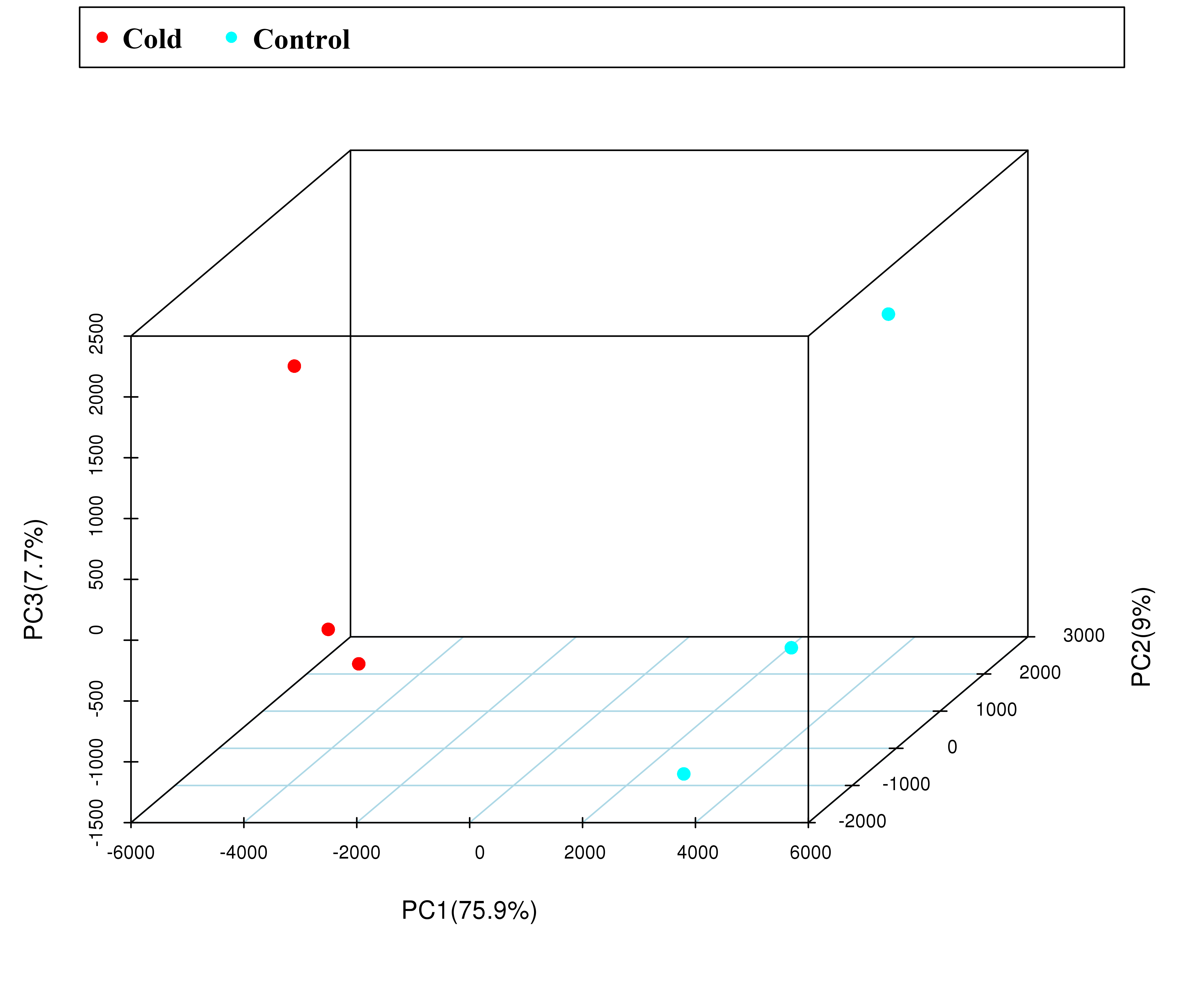

Supplement: Supplementary Figure 1 — Quality inspection of maize seedling root transcriptome data under low temperature stress. [file Image_1.TIF]

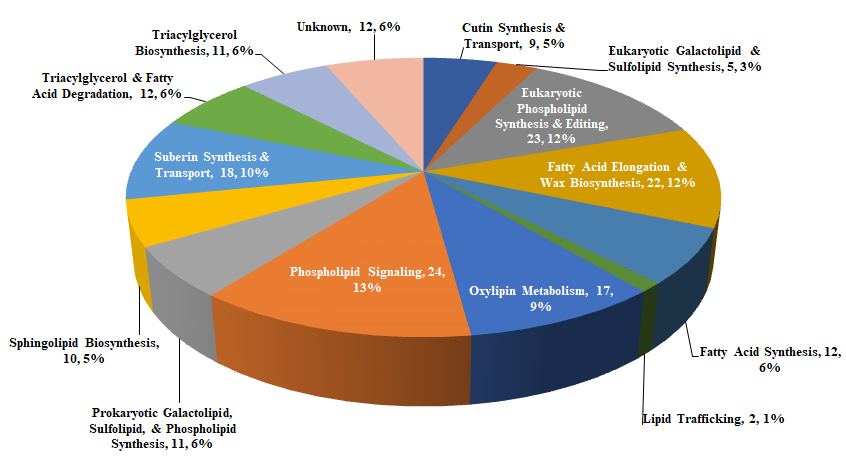

Supplement: Supplementary Figure 2 — Functional annotation of genes related to lipid metabolism in maize roots under low temperature stress. [file Image_2.TIF]

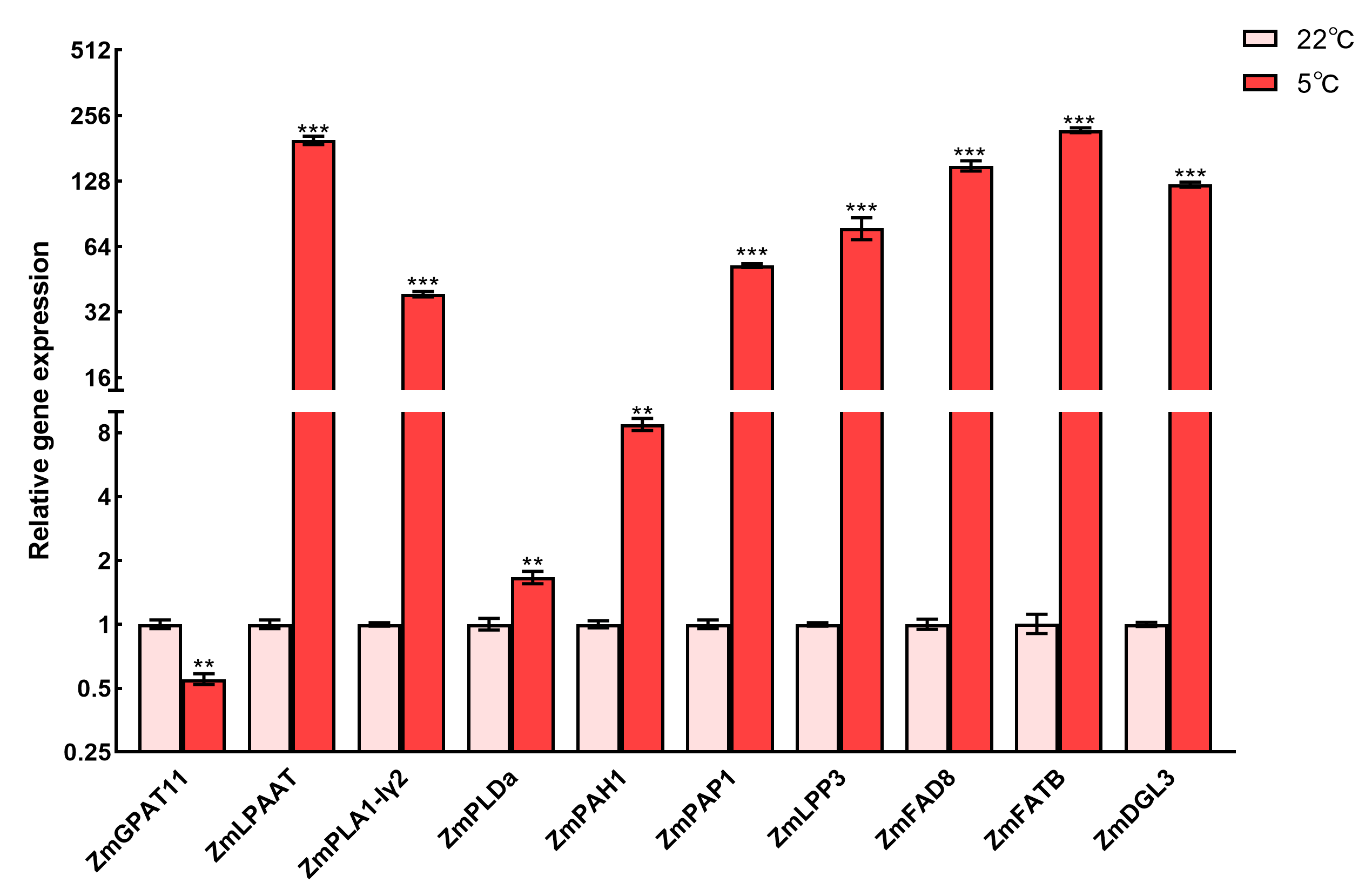

Supplement: Supplementary Figure 3 — Verification of differentially expressed genes in maize seedling roots under low temperature stress by qRT-PCR. [file Image_3.TIF]

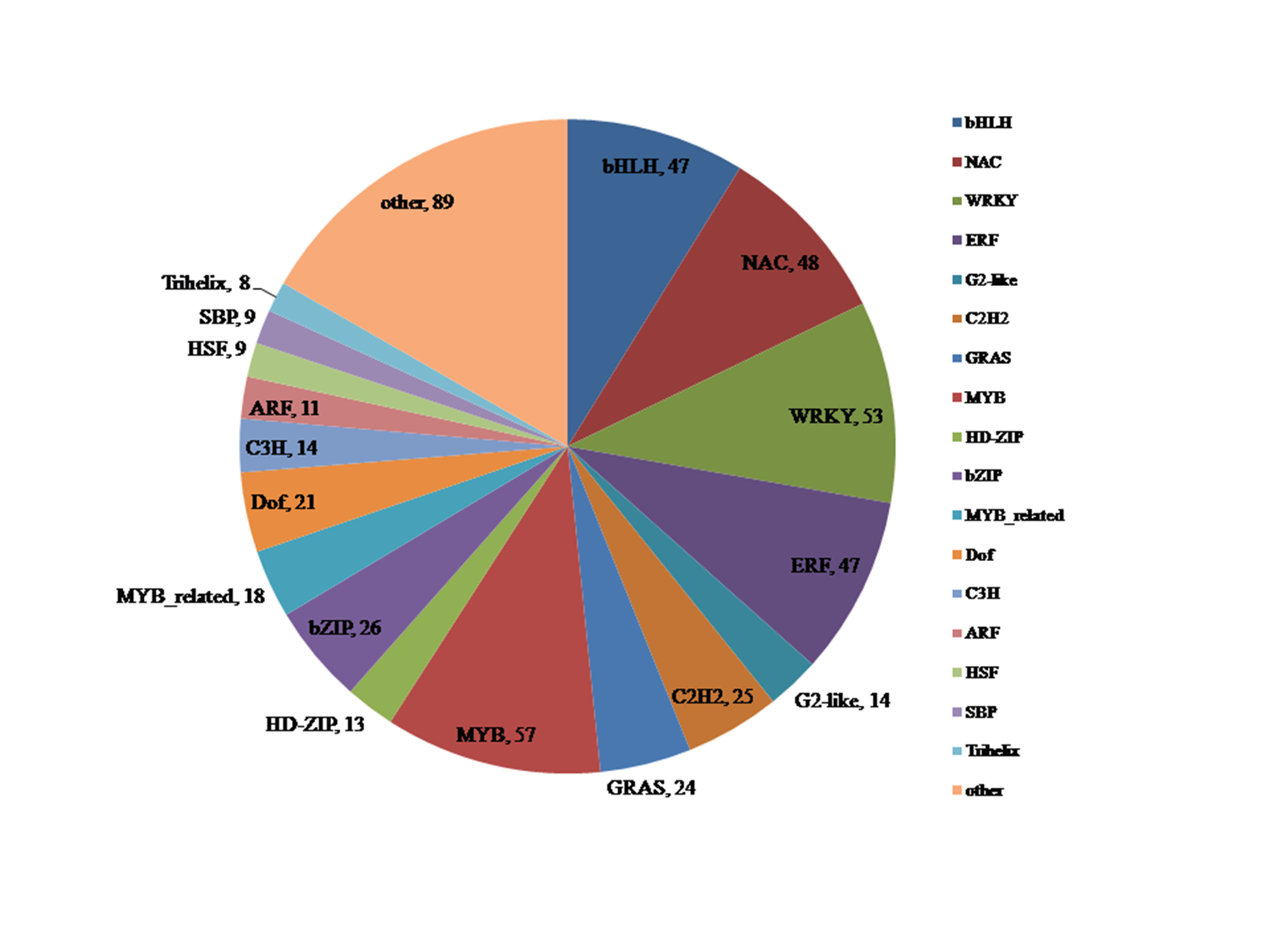

Supplement: Supplementary Figure 4 — Enrichment of transcription factors of genes related to lipid metabolism in maize roots under low temperature stress. [file Image_4.TIF]
